# Plant root suberin: A layer of defence against biotic and abiotic stresses

**DOI:** 10.3389/fpls.2022.1056008

**Published:** 2022-11-25

**Authors:** Anle Chen, Tong Liu, Zhou Wang, Xinping Chen

**Affiliations:** ^1^ Interdisciplinary Research Center for Agriculture Green Development in Yangtze River Basin, and College of Resources and Environment, Southwest University, Chongqing, China; ^2^ College of Plant Protection, Southwest University, Chongqing, China

**Keywords:** endodermis, suberization, plant lipophilic barriers, plasticity, stress tolerance

## Abstract

Plant roots have important functions, such as acquiring nutrients and water from the surrounding soil and transporting them upwards to the shoots. Simultaneously, they must be able to exclude potentially harmful substances and prevent the entry of pathogens into the roots. The endodermis surrounds the vascular tissues and forms hydrophobic diffusion barriers including Casparian strips and suberin lamella. Suberin in cell walls can be induced by a range of environmental factors and contribute to against biotic and abiotic threats. Tremendous progress has been made in biosynthesis of suberin and its function, little is known about the effect of its plasticity and distribution on stress tolerance. In field conditions, biotic and abiotic stress can exist at the same time, and little is known about the change of suberization under that condition. This paper update the progress of research related to suberin biosynthesis and its function, and also discuss the change of suberization in plant roots and its role on biotic and abiotic stresses tolerance.

## Introduction

Plant roots acquire nutrients and water from soil and transport them to the shoots, while toxic compounds must be restricted from entering the plant. To reach the central vasculature of the root, water and nutrients must cross the major tissue types of the root: the epidermis, the cortex and the endodermis. The epidermis is the outermost cell layer of young roots. The endodermis surrounds the vascular tissue. All vascular plants normally develop an endodermis in their roots, and the majority of angiosperm roots also have an exodermis which is a cell layer beneath the epidermis. The exodermis is absent in *Arabidopsis*, soybean, oats, barley and wheat (Perumalla et al., 1990; Thomas et al., 2007; Ranathunge et al., 2008; Kreszies et al., 2018).

The differentiation of the endodermis starts by the formation of the Casparian strip, as root development progresses, another type of barrier is formed, called suberin lamellae. This waxy, lipophilic layer is deposited all around the cellular surface of endodermal cells, impregnating the space between the cell wall and the plasma membrane (Nawrath et al., 2013; Vishwanath et al., 2015). The suberin layer appears to act as a transcellular barrier controlling the uptake or passive diffusion of ions from the apoplast into the symplastic environment of the endodermal cells (Barberon, 2017; Doblas et al., 2017). Plants have a variety of physiological response mechanisms when they are subjected to various stresses from the environment, and the deposition of suberin lamellae in the endodermis is one of them. This response can control the transport efficiency of various ions in plants, for reaching the ions balance in plants and reducing the toxic effect on plant growth and development. Suberin in cell walls can be induced by biotic and abiotic stresses (Hose et al., 2001; Enstone et al., 2003; Krishnamurthy et al., 2011; Tylova et al., 2017). It was also shown that suberin biosynthesis in Arabidopsis is affected by nutrient deficiencies such as iron (Fe), manganese (Mn), Zinc (Zn), potassium (K) and sulphur (S) (Barberon et al., 2016). It was suggested that plants can adapt to a sub-optimal nutrient supply in a highly dynamic and ion specific matter, either by an increasing or by a decreasing endodermal suberization (Barberon et al., 2016; Doblas et al., 2017). The suberized endodermis isolates the stele from the rest of the root, and function as a barrier to nematode entrance and against pathogen invasion into the xylem and spread throughout the plant (Holbein et al., 2019; Kashyap et al., 2021).

## Establishment of suberin lamellae in the cell wall

The endodermal differentiation is marked by the deposition of suberin lamellae, which cover the cellular surface of endodermal cells (Nawrath et al., 2013; Vishwanath et al., 2015). From the root tip to the base, the suberized endodermal cells first form a patchy zone, then a continuous zone (Geldner, 2013; Barberon et al., 2016). The fully suberized zone has some non-suberized cells, called passage cells, which are always located close to xylem poles. The establishment of passage cells is governed by repression of cytokinin signalling in the root meristem, which ultimately results in non-suberized endodermal cells (Andersen et al., 2018).

Suberin is a chemically complex heteropolymer, which is a glycerol-based polymer consisting of a polyaliphatic polyester linked with phenolic components (Kolattukudy, 1981; Franke and Schreiber, 2007; Pollard et al., 2008). Transmission electron microscopy (TEM) shows that the suberin lamellae contain electron-lucent and electron-dense contrasts, suggested to consist of polyaliphatics and polyaromatics, respectively (Graca and Santos, 2007). Suberin is chemically similar to cutin which is an insoluble lipid polyester deposited outside of the primary cell wall and which covers the outer face of the epidermal wall. However, suberin contains an aromatic domain which is not present in cutin (Schreiber, 2010).

The chemical composition of suberin in Arabidopsis roots was analysed by gas chromatography coupled to mass spectrometry (GC-MS), monounsaturated ω-hydroxyacids, α,ω-dicarboxylic acids and glycerol are the major monomers of suberin, followed by alcohols and unsubstituted fatty acids (Franke et al., 2005). Suberin consists of polyaliphatic domains and polyaromatic domains. It was suggested that the aliphatic domain primarily made suberin a transport barrier for water due to its high hydrophobicity (Zimmermann et al., 2000; Kreszies et al., 2018). In barley, the amount of aliphatic suberin in the primary root was increased in response to osmotic stress, and the osmotic stress-induced aliphatic suberin markedly reduced the water flow through the apoplast (Kreszies et al., 2019). In potato, the aromatic domain of suberin was reported to provide resistance to pathogen penetration (Lulai and Corsini, 1998), and it was suggested that the aromatic domains primarily make suberin as a transport barrier for nutrients (Kreszies et al., 2018). The composition of suberin between species is similar, however the content of suberin is strongly species dependent and can be induced by a range of environmental factors, which affect the efficiency of suberin as a barrier.

## Biosynthesis of suberin

The biosynthetic machinery responsible for suberin productions is complex due to the chemical diversity of the suberin polymers. Chemical analysis and biochemical studies three decades ago were the initial steps to elucidate the biosynthesis pathways and structure of suberin (Kolattukudy, 1981). After that, approaches of reverse genetics on bark, potato periderm, cotton fibres, Arabidopsis root endodermis and seed coats made further progress on suberin biosynthesis (Molina et al., 2009; Ranathunge et al., 2011; Beisson et al., 2012; Li-Beisson et al., 2013; Graca, 2015). However, the sequence of biosynthetic reactions, transport mechanism of monomers and the site of polymerization of the precursors remain to be elucidated.

**Figure 1:**
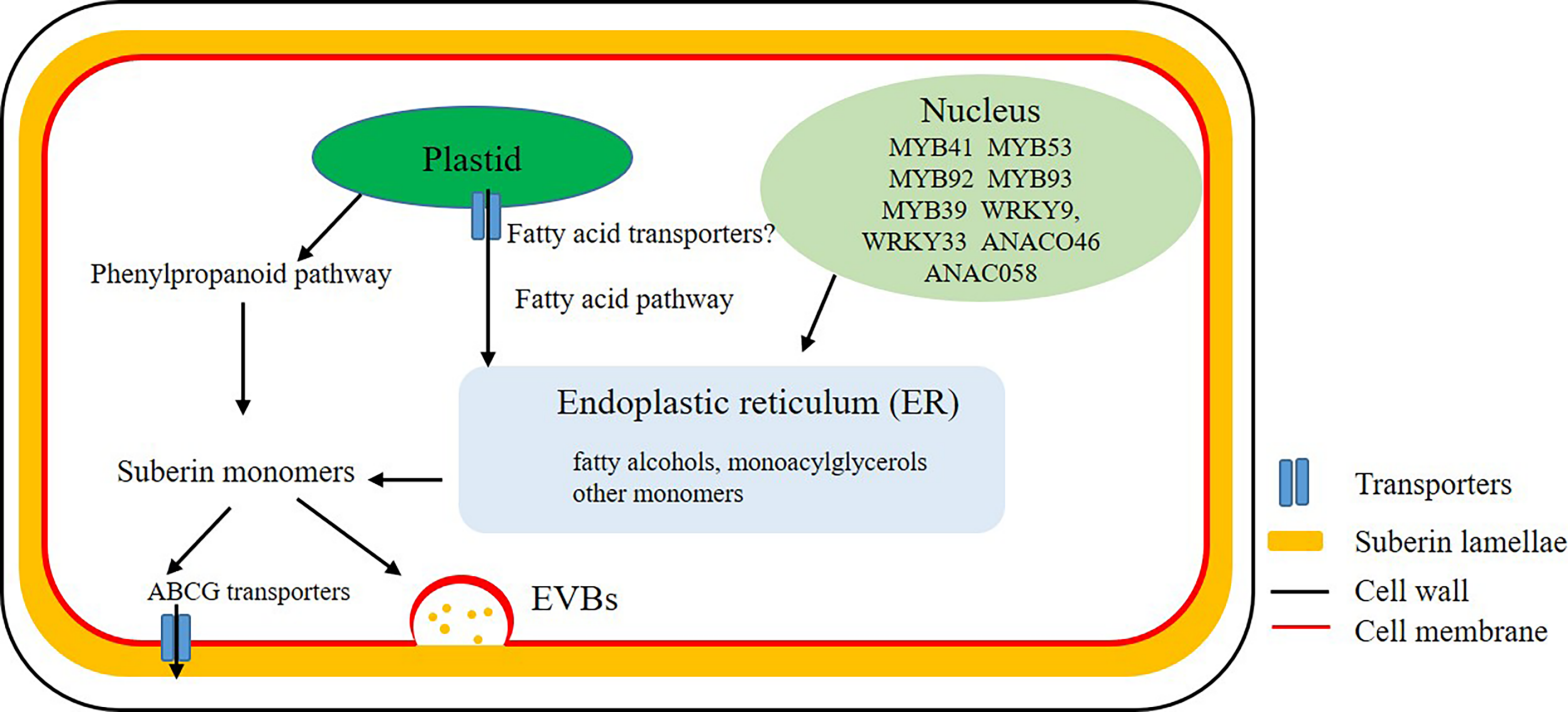
Biosynthesis and transport of suberin. Mostly biosynthesis of suberin monomers take place at the endoplasmic reticulum (ER). After a series of complex enzyme-induced reactions, fatty acids and other lipids in plastid are transported to the ER to synthesize fatty alcohols, monoacylglycerols and other monomers. Monomers, oligomers or polymers are probably transported cross the plasma membrane by ATP-binding cassette (ABC) transporters or Extracellular Vesiculo-tubular containing Bodies (EVBs).

Biosynthesis of suberin monomers involves fatty acid and phenylpropanoid pathways. In plants, fatty acid synthesis occurs in the plastid stroma. However, the relationship between regulation of diffusion or transport of fatty acids through the lipid membrane of plastid with suberin biosynthesis is not clear (**Figure 1**). The core reactions of the suberin biosynthetic pathway were believed to take place at the endoplasmic reticulum (ER) (**Figure 1**) (Li-Beisson et al., 2013). A large number of genes encoding enzymes involved in the synthesis of suberin have been identified (Vishwanath et al., 2015). However, many aspects of suberin biosynthesis remain undetermined. Whether suberin precursors are exported as monomers or building blocks is unclear (Beisson et al., 2012). The ATP-binding cassette **(**ABC) transporters, lipid transport proteins and secretion through vesicles are thought to be involved in the export of these monomers or building blocks out of plasma membrane to the site of polymerization (**Figure 1**) (De Bellis et al., 2022). The transporters that transport aliphatic monomers out of the plasma membrane have recently been identified, and all of them belong to the ABCG subfamily (Do et al., 2018). In Arabidopsis root, proteins of AtABCG2, AtABCG6 and AtABCG20 make contribution for the formation of suberin lamellae in endodermis (Yadav et al., 2014). Other ABC transporters, like OsABCG5 in rice root and StABCG1 in potato root and tuber periderm were also identified and described as suberin monomer transporters (Landgraf et al., 2014; Shiono et al., 2014).

Recently some regulatory genes of suberin synthesis have been described. In Arabidopsis, the transcription factorAtMYB41, AtMYB53, AtMYB92 and AtMYB93 are positive regulators of suberin biosynthesis in roots (Shukla et al., 2021). AtMYB107, AtMYB9 are required for suberin assembly in the Arabidopsis seed coat which were revealed highly conserved in angiosperms (Lashbrooke et al., 2016; Gou et al., 2017) and MdMYB93 was also described as a regulator of suberin deposition in apple fruit skins (Legay et al., 2016).

## Deposition of suberin in cell wall

Suberin separates the cell wall from the plasma membrane and it can be found in root endodermis/hypodermis, seed coats, bark, and potato tuber skin (Schreiber, 2010). Suberin also deposits at the wound edges of potato periderm (Lulai et al., 2008) and at the site of lateral root emergence, where the CSs are disrupted (Li et al., 2017). The distribution of suberin in different plant tissues suggests that plants can synthesise and deposit suberin whenever and wherever they need to form a barrier (Kolattukudy, 2001; Franke and Schreiber, 2007). Recently, it is report that GELPs (GDSL-type Esterase/Lipase Protein family) play a key role in suberin polymerization and degradation both in the context of lateral root emergence and endodermal layer (Ursache et al., 2021). However, the mechanisms that regulate the onset of suberization are unclear.

The deposition of suberin in Arabidopsis roots starts in a patchy manner, then more and more endodermal cells are suberized until the whole endodermis is suberized except for some passage cells located close to xylem poles (Geldner, 2013). Suberization patterns in other plant species were also reported, there is variation between plant species due to the difference of root anatomy (Thomas et al., 2007; Waduwara et al., 2008; Ranathunge et al., 2017). In barley from root tip to root base, the deposition of suberin in endodermis was described as four different zones including non-suberized zone, patchy zone, continuous zone and fully suberized zone, and fully suberized zone was always the longest zone, accounting for about 50% of the root length, followed (in length) by the continuous, non-suberized and lastly the patchy zone (Chen, et al., 2019).

## Abiotic and biotic stress tolerance

Besides the function as a barrier for water and nutrients, suberin lamellae in plant roots also contribute to abiotic and biotic stress tolerance. It has been found that suberization is induced during salt stress, cadmium (Cd) toxicity and ammonium stress, which suggest that the function of suberin is to block the entry of toxic elements into the cytoplasm (Krishnamurthy et al., 2009; Liska et al., 2016; Ranathunge et al., 2016). Suberization is also induced under drought and waterlogged conditions, suggesting a role for suberin in preventing water and oxygen loss (Kotula et al., 2009; Shiono et al., 2014; Liska et al., 2016). In rice, suberization is enhanced during salt stress and the extent of suberin deposition in the primary roots is negatively correlated with Na uptake into the shoots as is also the case for hydraulic (Krishnamurthy et al., 2009; Krishnamurthy et al., 2011). In Arabidopsis, *horst* and *gpat5* mutants with reduced suberization show increased water transport and higher sensitivity to salt stress (Beisson et al., 2007; Ranathunge and Schreiber, 2011). In Arabidopsis, it was shown that suberization was reduced under Fe, Mn and Zn deficiencies, whereas S and K deficiencies lead to enhanced suberin (Barberon et al., 2016). The decrease in suberin was shown to be mediated by ethylene, whereas the increased suberization was controlled by abscisic acid (ABA). This endodermal suberin plasticity may reflect an adaptation of plant roots to cope with fluctuating nutrient availability by modulating the uptake of Fe, Mn and Zn and retain K and S in the stele (Barberon, 2017; Doblas et al., 2017). In the primary axis of barley roots, it was found that the suberization also responds to Mn deficiency, thus confirming the results from Arabidopsis. However, this response is not a linear process, as suberization was first reduced during mild Mn deficiency and then enhanced during strong Mn deficiency. Since the reduced suberin has secondary effects on the uptake of other nutrient elements, such as promoting K leakage from the stele, the enhanced suberization during strong Mn deficiency might favour retainment of K in the stele, thus maintaining nutrient homeostasis (Chen, et al., 2019).

**Figure 2:**
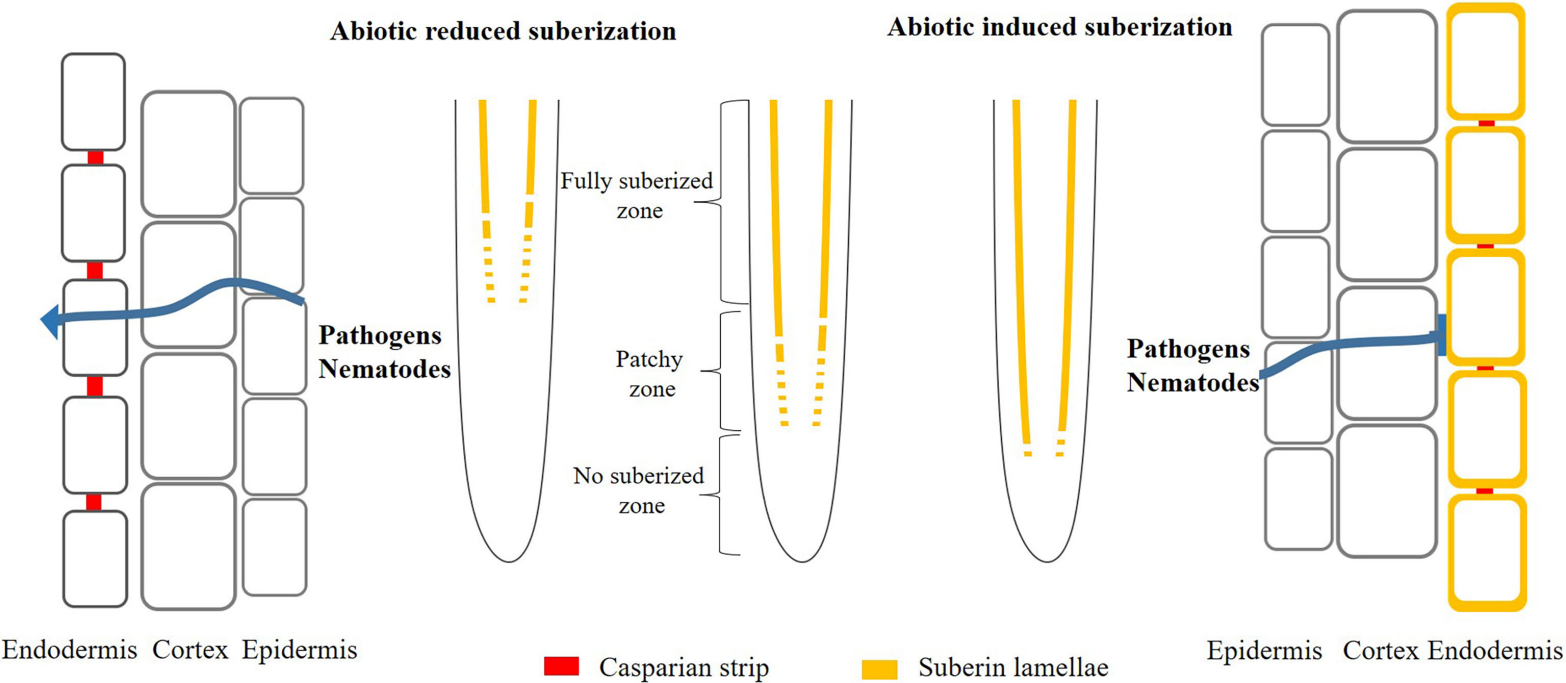
Model of abiotic induced/reduced suberization and its effect on pathogens and nematodes infection plants. Under normal conditions, plant roots deposit suberin in the endodermis. However, once the plant is stimulated by extern environment (nutrient deficiency or toxicity), it will deposit more or less suberin in the endodermis, thus protecting the plant from extern damage. Simultaneously, the change of suberization probably promote (decreased suberin) or prevent (enhanced suberin) the entry of pathogens and nematodes into the roots.

Moreover, suberin might act as a barrier to prevent penetration by pathogens and nematode (**Figure 2**). The suberized endodermis serves as a line of defense preventing pathogens from invading the vascular tissues and spread throughout the plant (Ranathunge et al., 2008; Holbein et al., 2019; Kashyap et al., 2022). In soybean, it has been shown that there is a strong correlation between the exent of suberin and resistance to the fungus (Thomas et al., 2007). To colonize the root vasculature, the fungal hyphae have to penetrate the suberized layers and it was found that it took more time for hyphae growth in cultivars with high content of suberin (Ranathunge et al., 2008). Suberin also play important role in beneficial biotic interactions and the coordination between root suberin and the microbiome leads to a balancing of the plant ionome, which make plants adapt to abiotic stress condition (Salas-Gonzalez et al., 2020).

## Perspectives

The growth of the human population will increase the demand for food in the future. However, the change of climate will intensify extreme weather conditions, for example drought, which might lead to decreased crop production. It is important to develop crops with increased yield and these crops should also be adapted to the specific soil conditions and climatic environment. The CSs and suberin lamellae in the endodermis of plant roots seem to play pivotal roles in controlling the uptake of water and nutrients and protect plants against biotic and abiotic threats (Barberon, 2017; Doblas et al., 2017; Kreszies et al., 2018). Understanding of the mechanisms underlying the functions of these root barriers is important, as this knowledge might help to develop crop varieties with improved nutrient use efficiency and better tolerance towards e.g. nutrient deficiencies, drought, salt stress and pathogens.

Suberin is not only deposited in the endodermis of plant roots but also in the bundle sheath of plant leaves, the vascular tissues in roots and leaves are thus surrounded by suberized cells. As a transcellular barrier, suberin has a similar function both in root and leaves, which affects the fluxes of solutes and pathogen penetration. Since the vascular tissues are continuous in the whole plant, it is necessary to analyse the function of suberin as a barrier in leaves and roots at the same time. Thereby, better understanding of the function of suberin as a barrier can be obtained. Finally, the biotic and abiotic (nutrient deficiency or toxic) stress can exist at the same time in the field condition. It is necessary to analysis the effect of suberization on pathogen penetration under abiotic condition.

## Author contributions

AC drafted and revised the manuscript. TL, ZW and XC revised the manuscript. All authors contributed to the article and approved the submitted version.

## Funding

This work was supported by the China Agriculture Research System (CARS-02), the natural Science Foundation of Chongqing (4312100205), China Postdoctoral Science Foundation (2020M673105) and Innovation and Entrepreneurship Program for College Students (Southwest University, X202210635025).

## Conflict of interest

The authors declare that the research was conducted in the absence of any commercial or financial relationships that could be construed as a potential conflict of interest.

## Publisher’s note

All claims expressed in this article are solely those of the authors and do not necessarily represent those of their affiliated organizations, or those of the publisher, the editors and the reviewers. Any product that may be evaluated in this article, or claim that may be made by its manufacturer, is not guaranteed or endorsed by the publisher.
